# Association between atherogenic index of plasma and gestational diabetes mellitus: a prospective cohort study based on the Korean population

**DOI:** 10.1186/s12933-024-02341-9

**Published:** 2024-07-05

**Authors:** Juan Zhang, Yaoyu Suo, Li Wang, Dong Liu, Yue Jia, Yajuan Fu, Weining Fan, Yideng Jiang

**Affiliations:** 1 School of Basic Medicine, Ningxia Medical University, Yinchuan, China; 2 General Hospital of Ningxia Medical University, Yinchuan, China; 3 National Health Commission Key Laboratory of Metabolic Cardiovascular Diseases Research, Ningxia Medical University, Yinchuan, China

**Keywords:** Gestational diabetes mellitus, Predictors, Atherogenic index of plasma, Cohort study

## Abstract

**Background:**

Atherogenic index of plasma (AIP) is a non-traditional lipid parameter that can reflect the burden of atherosclerosis. A lipid profile resembling atherosclerosis emerged during pregnancy. Although lipid metabolism is pivotal in diabetes pathogenesis, there is no evidence linking AIP to gestational diabetes mellitus (GDM). Therefore, our objective was to explore the relationship between AIP and GDM and assess AIP's predictive capability for GDM.

**Methods:**

This was a secondary analysis based on data from a prospective cohort study in Korea involving 585 single pregnant women. AIP was calculated as log10 (TG/HDL). We examined the relationship between AIP and GDM using logistic regression models, curve fitting, sensitivity analyses, and subgroup analyses. Receiver operating characteristic (ROC) analysis was also used to determine the ability of AIP to predict GDM.

**Results:**

The average age of the participants was 32.06 ± 3.76 years. The AIP was 0.24 ± 0.20 on average. The GDM incidence was 6.15%. After adjustment for potentially confounding variables, AIP showed a positive linear relationship with GDM (*P* for non-linearity: 0.801, OR 1.58, 95% CI 1.27–1.97). The robustness of the connection between AIP and GDM was demonstrated by sensitivity analyses and subgroup analyses. An area under the ROC curve of 0.7879 (95% CI 0.7087–0.8671) indicates that AIP is an excellent predictor of GDM. With a specificity of 75.41% and sensitivity of 72.22%, the ideal AIP cut-off value for identifying GDM was 0.3557.

**Conclusions:**

This study revealed that the AIP at 10–14 weeks of gestation was independently and positively correlated with GDM risk. AIP could serve as an early screening and monitoring tool for pregnant women at high risk of GDM, thereby optimizing GDM prevention strategies.

**Trial registration:**

ClinicalTrials.gov registration no. NCT02276144.

**Graphical abstract:**

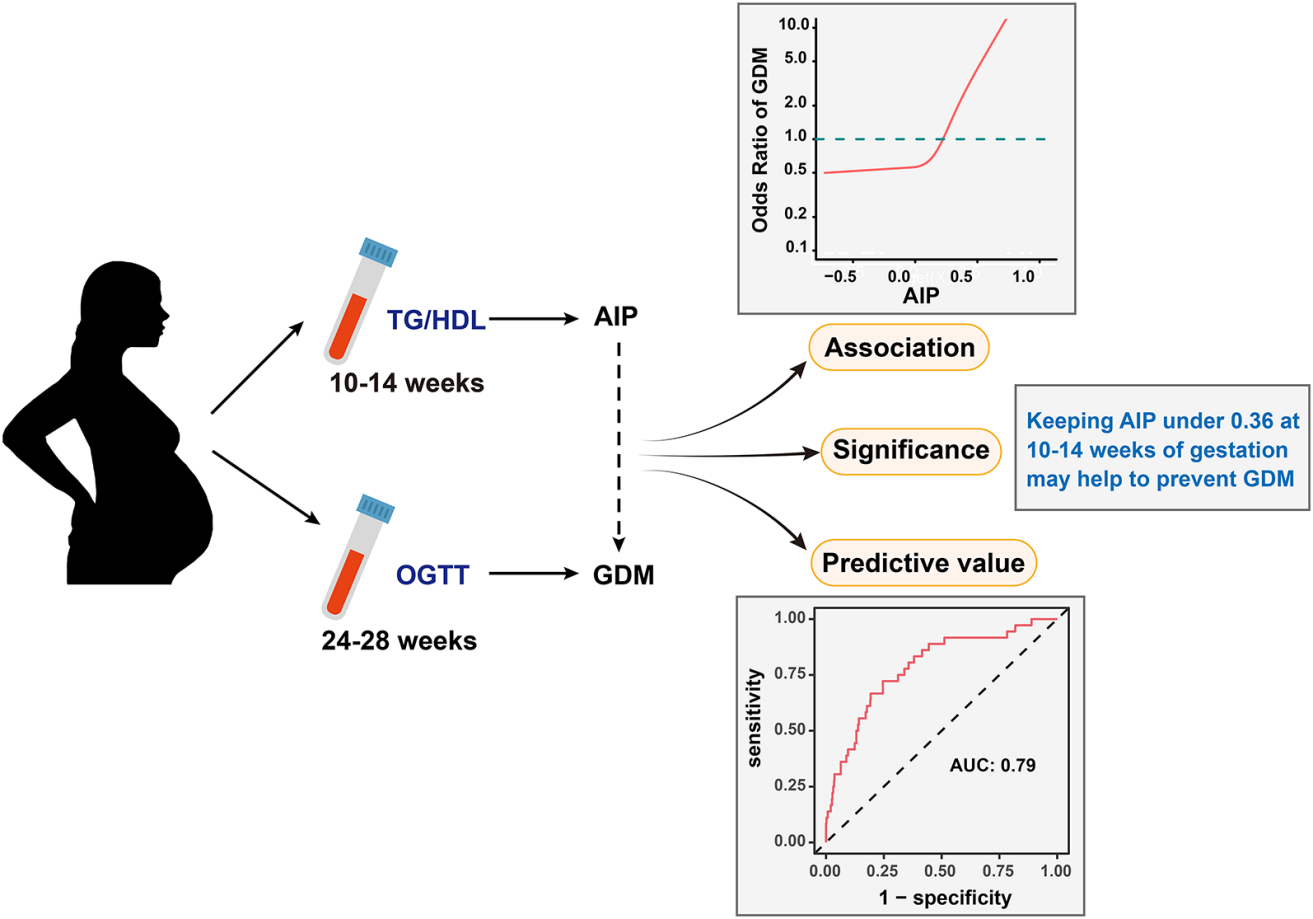

**Supplementary Information:**

The online version contains supplementary material available at 10.1186/s12933-024-02341-9.

## Significance statement

Our study revealed that AIP is an independent risk factor for GDM, and is capable of early and effective prediction of GDM, which is particularly valuable for pregnant women with normal TG and HDL levels who often overlook lipid management.

## Introduction

Gestational diabetes mellitus (GDM) refers to abnormal glucose tolerance with onset or first recognition during pregnancy [1]. It is currently the most common complication of pregnancy, affecting approximately 15% of pregnancies worldwide [2]. Due to epidemiological factors, including increased rates of obesity in women of reproductive age and rising maternal age, the incidence of GDM has consistently risen globally in recent decades [3]. GDM carries risks for the mother, fetus, and neonate. According to the Hyperglycemia and Adverse Pregnancy Outcome (HAPO) research, the risk of unfavorable outcomes for mothers, fetuses, and newborns steadily increases as maternal glycemia increases [4]. Additionally, GDM is recognized as a substantial risk factor for cardiovascular diseases and diabetes in mothers [5], and it is also linked to obesity and impaired insulin sensitivity in their children [6]. The clinical diagnosis of GDM is usually at 24 and 28 weeks of gestation [7]. However, at this stage, it is possible that both the mother and fetus have already experienced negative effects to different extents [8, 9]. Therefore, identifying high-risk women with GDM promptly is crucial to reducing adverse outcomes and halting intergenerational metabolic issues.

The atherogenic index of plasma (AIP), introduced by Dobiásová and Frohlich in 2001, is an innovative lipid indicator calculated from the logarithm of the triglyceride (TG) to high-density lipoprotein cholesterol (HDL) ratio, serving as an effective parameter for atherosclerosis [10]. Recent research has consistently confirmed the connection between AIP and diabetes [11, 12], as well as AIP and prediabetes [13]. However, few studies have been performed to determine whether AIP is linked to GDM, and the potential of using AIP in early pregnancy to predict GDM. Therefore, our research focused on exploring the precise connection between AIP and GDM, seeking a simple and practical clinical indicator for GDM's early identification.

## Methods

### Study design

A prospective cohort study design was utilized in this study. The dataset was sourced from an existing research database, the "Fatty Liver in Pregnancy" registry (ClinicalTrials.gov registration no. NCT02276144), established by Korean researchers [14]. They prospectively enrolled singleton pregnant women presenting for prenatal care before 14 weeks of gestation at Incheon Seoul Women’s Hospital and Seoul Metropolitan Government Seoul National University Boramae Medical Center to determine the risk of nonalcoholic fatty liver disease (NAFLD) on pregnancy outcome.

### Data source

The raw data used in this research were obtained freely from the article "Nonalcoholic fatty liver disease is a risk factor for large-for-gestational-age birthweight" by Lee et al. in PLoS ONE (https://journals.plos.org/plosone) [15]. They have been published under the terms of the Creative Commons Attribution License, permitting freely used, distributed, and reproduced in any format on the condition that proper attribution is provided to the author and source. We are thankful to the individuals who provided data for their priceless contributions.

### Study population

The original research was approved by the Institutional Review Board at Seoul National University Boramae Medical Center and the Public Institutional Review Board of the Korean Ministry of Health and Welfare. As a result, there was no need for ethical approval of this secondary analysis. Before enrollment, each participant filled out and signed a consent form to participate. Additionally, the original study complied with the principles outlined in the Declaration of Helsinki, and our secondary analysis also used the STROBE guidelines, every step followed the pertinent guidelines and rules.

**Fig. 1:**
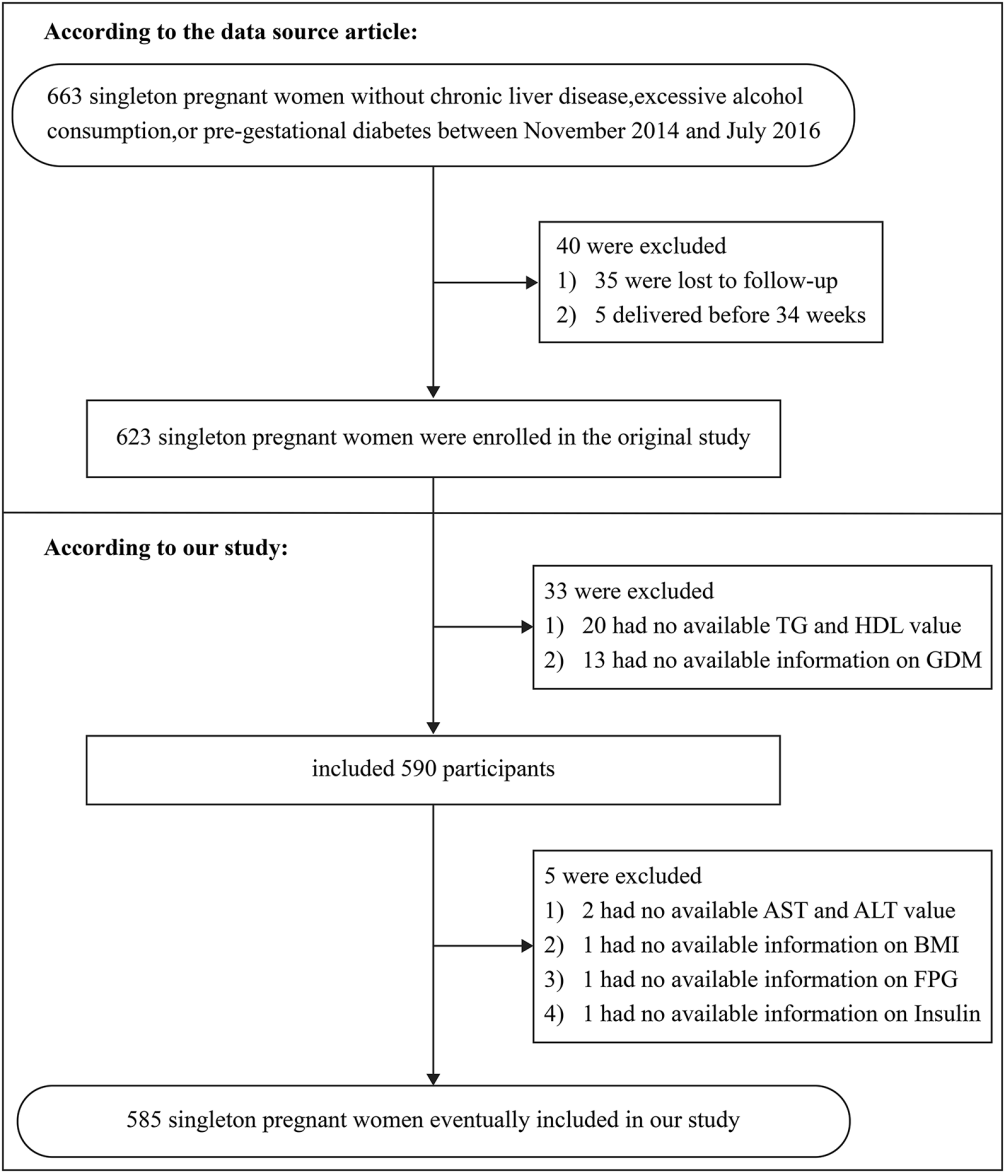
Flowchart of study participants

The original study initially recruited 663 singleton pregnant women who did not have chronic liver disease, excessive alcohol consumption, or pre-gestational diabetes. However, 40 individuals were eliminated because of missing follow-up or because they experienced a premature delivery prior to 34 weeks. As a result, the total number of individuals involved in the initial research was 623. According to our research design, we excluded an additional 33 individuals because they had missing data on the exposure variables (TG and HDL, *n* = 20) or outcome variable (GDM, *n* = 13). Next, we excluded 5 individuals whose covariate data were missing (*n* = 5). Ultimately, our study included 585 singleton pregnant women (Fig. 1).

### Data collection

Data was collected and sorted by skilled healthcare professionals. A standardized survey was employed to collect and document general clinical and demographic details, such as the age of the mother, nulliparity, pre-pregnancy body mass index (BMI), lifestyle habits (alcohol consumption), and diabetes history. Venous blood samples were obtained at 10–14 weeks of gestation after fasting for at least 8 h to assess hematological markers, including aspartate aminotransferase (AST), alanine aminotransferase (ALT), gamma-glutamyl transferase (GGT), total cholesterol (TC), TG, HDL, low-density lipoprotein cholesterol (LDL), fasting plasma glucose (FPG), and insulin levels. Liver ultrasound was used to assess the presence of NAFLD. At 24–28 weeks of gestation, every participant was tested for GDM with the two-step screening test [16, 17]. For more in-depth details regarding the data collection and variable definitions, refer to the original research [15].

### Variables

#### Exposure variable and outcome variable

The AIP, calculated as log10 (TG/HDL) [10], served as the exposure variable. Participants were divided into three categories based on the AIP tertiles: Q1 (< 0.16), Q2 (0.16–0.32), and Q3 (> 0.32). The outcome variable was GDM.

#### Covariates

Covariates were chosen for our study by referencing the initial research, medical expertise, and literature on GDM-related risk factors. Consequently, the following variables were chosen as covariates: age, BMI, AST, ALT, GGT, TC, LDL, NAFLD, FPG, insulin, and homeostasis model assessment-insulin resistance (HOMA-IR). The formula for calculating HOMA-IR was [FPG (mmol/L) × insulin (μU/mL)/22.5], following established methodologies [18].

### Statistical analysis

The mean and standard deviation (SD) were used for continuous variables with a normal distribution, while the median and interquartile range (IQR) were used for a skewed distribution. Frequencies (%) were used to describe categorical variables. To assess the variations among different groups, we conducted one-way analyses of variance for a normal distribution, Kruskal–Wallis tests for a skewed distribution, and chi-square tests for categorical variables. We used logistic regression models to calculate the OR and 95% CIs for the AIP-GDM connection. Model 1 was an unadjusted rough model; Model 2 was adjusted for age, BMI, and nulliparity. Model 3 was further adjusted for AST, ALT, and GGT. Model 4 was additionally adjusted for FPG, insulin, and HOMA-IR. Model 5 was fully adjusted, built upon Model 4, and further adjusted for TC, LDL, and NAFLD. Besides, curve fitting by restricted cubic splines (RCS) were used to assess linearity between AIP and GDM, as well as the dose–response relation after adjusting variables in Model 5. Furthermore, homogeneity among subgroups was evaluated by a stratified logistic regression model that took into account age, pre-pregnancy BMI, nulliparity, HOMA-IR, and NAFLD. Ultimately, likelihood ratio tests were used to examine the associations between AIP and GDM in the subgroups. In order to assess the reliability of our findings, participants with a pre-pregnancy BMI ≥ 25 kg/m^2^ or NAFLD were removed from the sensitivity testing. Finally, we created a receiver operating characteristic (ROC) curve to assess how well AIP and other indicators can predict GDM by determining the area under the curve (AUC) of the ROC curve and the most effective threshold.

As the sample size was decided solely by the provided data, there were no prior statistical power estimates conducted. R Statistical Software (version 4.2.2, http://www.R-project.org, The R Foundation) and the Free Statistics analysis platform (version 1.9, Beijing, China, http://www.clinicalscientists.cn/freestatistics) were used for all analyses. The tests were two-sided, with a *P* value < 0.05 indicating statistical significance.

## Results

### Characteristics of participants

This study involved 585 eligible pregnant women. Their basic characteristics did not differ significantly from those of the excluded population (Supplementary Table S1). Their average age was 32.06 ± 3.76 years. The mean AIP was 0.24 ± 0.20. 36 women (6.15%) were diagnosed with GDM at 24–28 weeks of gestation. Table 1 presents the baseline characteristics of the study population according to the AIP tertiles. It shows that in the Q3 group, participants generally had a higher incidence of GDM and NAFLD; higher levels of BMI, GGT, TG, LDL, insulin, and HOMA-IR; and lower HDL levels.

**Table 1:** The baseline characteristics of participants

Characteristics	AIP tertile			*P*-value
	Q1 (< 0.16)	Q2 (0.16–0.32)	Q3 (> 0.32)	
Participants	195	195	195	
Age (years)	31.52 ± 3.52	32.48 ± 3.56	32.17 ± 4.13	0.037
BMI (kg/m^2^)	21.22 ± 2.97	22.06 ± 3.55	22.78 ± 3.75	< 0.001
Nulliparity				0.054
No	116 (59.49%)	94 (48.21%)	97 (49.74%)	
Yes	79 (40.51%)	101 (51.79%)	98 (50.26%)	
AST (IU/L)	16.00 (14.00–18.50)	16.00 (14.00–19.00)	17.00 (14.00–21.00)	0.159
ALT (IU/L)	11.00 (8.00–13.50)	11.00 (8.00–15.00)	12.00 (8.00–18.00)	0.103
GGT (IU/L)	11.00 (9.50–14.00)	12.00 (10.00–15.00)	13.00 (10.00–17.00)	0.002
TC (mg/dL)	171.24 ± 26.79	171.79 ± 26.20	175.49 ± 28.22	0.243
TG (mg/dL)	81.79 ± 19.21	110.65 ± 21.58	163.95 ± 49.77	< 0.001
HDL (mg/dL)	74.14 ± 11.81	64.95 ± 10.89	55.81 ± 11.19	< 0.001
LDL (mg/dL)	80.74 ± 21.22	84.72 ± 20.02	86.50 ± 23.42	0.027
NAFLD				< 0.001
No	171 (87.69%)	168 (86.15%)	136 (69.74%)	
Yes	24 (12.31%)	27 (13.85%)	59 (30.26%)	
FPG (mg/dL)	76.93 ± 10.25	76.77 ± 8.62	77.20 ± 10.25	0.907
Insulin (μIU/mL)	6.40 (4.30–9.55)	7.90 (5.50–10.85)	10.60 (7.45–15.30)	< 0.001
HOMA-IR	1.20 (0.80–1.90)	1.50 (1.00–2.20)	2.00 (1.40–2.85)	< 0.001
GDM				< 0.001
No	192 (98.46%)	188 (96.41%)	169 (86.67%)	
Yes	3 (1.54%)	7 (3.59%)	26 (13.33%)	

Values were expressed as mean (standard deviation) or median (interquartile range) or *n* (%)

AIP, atherogenic index of plasma; BMI pre-pregnancy body mass index; AST, aspartate aminotransferase; ALT, alanine aminotransferase; GGT, gamma-glutamyl transferase; TC, total cholesterol; TG, triglyceride; HDL, high-density lipoprotein cholesterol; LDL, low-density lipid cholesterol; NAFLD, nonalcoholic fatty liver disease; FPG, fasting plasma glucose; HOMA-IR, homeostasis model assessment-insulin resistance; GDM, gestational diabetes mellitus

### Association between AIP and GDM

Table 2 displays the univariate analysis results. Pre-pregnancy BMI, ALT, GGT, FPG, insulin, HOMA-IR, TG, NAFLD, and AIP all showed a positive correlation with the risk of GDM, while HDL had an inverse association with GDM risk.

**Table 2:** The results of univariate analysis

Variable	Non-GDM (*n* = 549)	GDM (*n* = 36)	OR (95%CI)	*P*-value
Age (years)	32.03 ± 3.77	32.56 ± 3.61	1.04 (0.95–1.14)	0.413
BMI (kg/m^2^)	21.77 ± 3.21	25.81 ± 5.17	1.27 (1.17–1.38)	< 0.001
Nulliparity				
No	288 (52.46%)	19 (52.78%)	Ref	
Yes	261 (47.54%)	17 (47.22%)	0.99 (0.50–1.94)	0.970
AST (IU/L)	16.00 (14.00–19.00)	17.50 (15.00–21.25)	1.02 (0.99–1.05)	0.175
ALT (IU/L)	11.00 (8.00–14.00)	15.00 (10.00–25.50)	1.04 (1.01–1.06)	0.002
GGT (IU/L)	12.00 (10.00–15.00)	15.50 (11.00–23.00)	1.03 (1.01–1.06)	0.012
FPG (mg/dL)	76.47 ± 9.05	84.58 ± 15.22	1.07 (1.04–1.10)	< 0.001
Insulin (μIU/mL)	8.10 (5.30–11.20)	15.30 (8.88–21.90)	1.12 (1.07–1.17)	< 0.001
HOMA-IR	1.50 (0.90–2.20)	2.90 (1.67–4.68)	1.48 (1.22–1.78)	< 0.001
TC (mg/dL)	172.36 ± 26.88	180.22 ± 29.74	1.01 (1.00–1.02)	0.093
TG (mg/dL)	115.04 ± 41.69	176.11 ± 83.09	1.02 (1.01–1.02)	< 0.001
HDL (mg/dL)	65.37 ± 13.25	58.86 ± 16.46	0.96 (0.94–0.99)	0.005
LDL (mg/dL)	83.99 ± 21.19	84.02 ± 28.74	1.00 (0.98–1.02)	0.992
NAFLD				
No	459 (83.61%)	16 (44.44%)	Ref	
Yes	90 (16.39%)	20 (55.56%)	6.37 (3.18–12.78)	< 0.001
AIP	0.23 ± 0.19	0.46 ± 0.24	AIP*10: 1.77 (1.47–2.13)	< 0.001

Values were expressed as mean (standard deviation) or median (interquartile range) or *n* (%)

OR, odds ratio; CI, confidence interval; Ref, Reference; other abbreviations as in Table 1

**Fig. 2:**
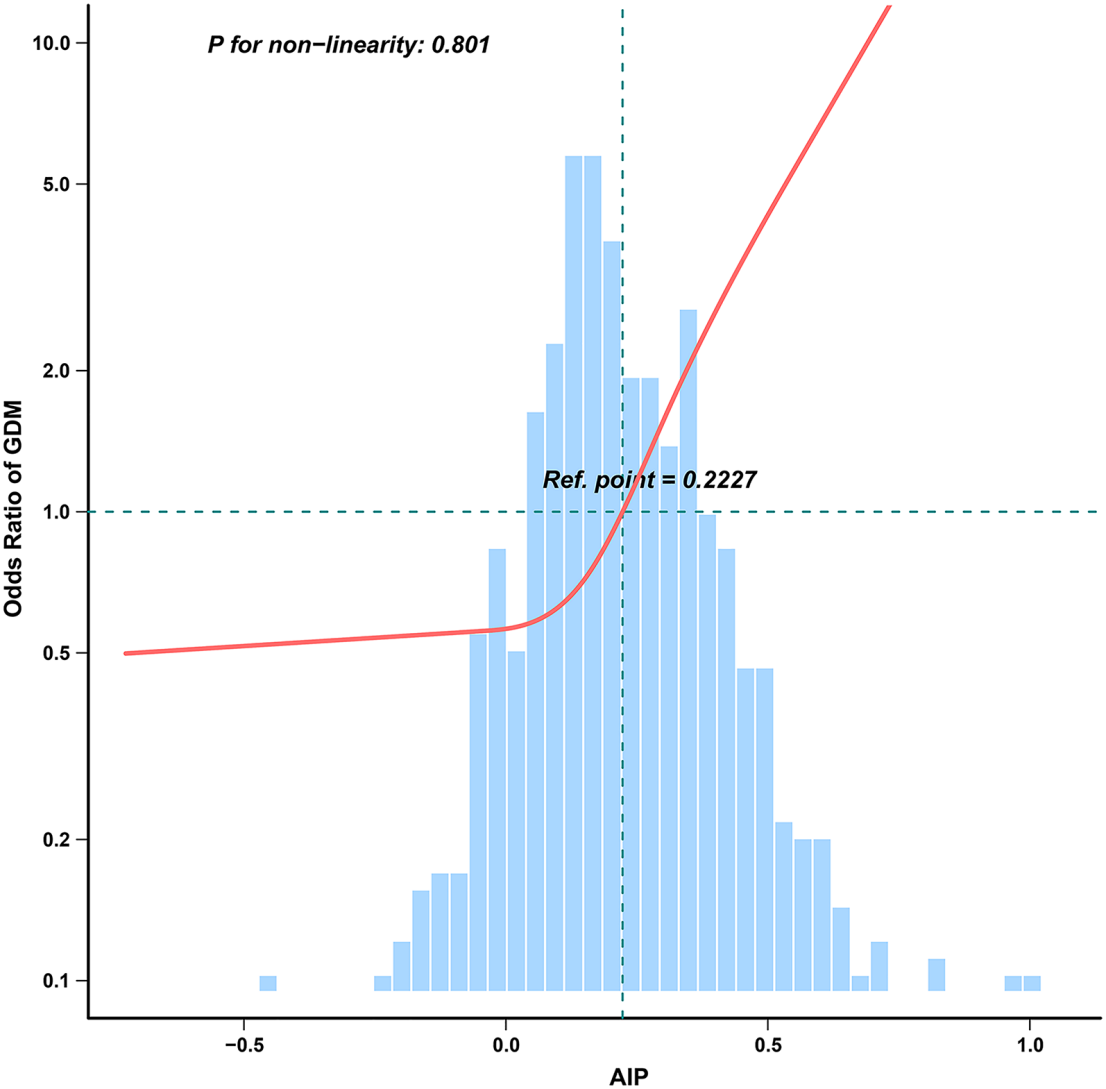
Association between AIP and GDM risk in RCS. Adjusted for Age + BMI + Nulliparity + AST + ALT + GGT + FPG + Insulin + HOMA-IR + TC + LDL-c + NAFLD

Table 3 shows an independently positive correlation between AIP and the risk of GDM across all multivariable logistic regression models, regardless of confounding variable adjustments (Model 1: OR 1.77, 95% CI 1.47–2.13; Model 2: OR 1.69, 95% CI 1.39–2.05; Model 3: OR 1.67, 95% CI 1.37–2.04; Model 4: OR 1.65, 95% CI 1.33–2.04; Model 5: OR 1.58, 95% CI 1.27–1.97). The confidence interval distribution provides additional evidence for the independent relationship between AIP and GDM risk. It is noteworthy that, after accounting for possible influencing factors (Model 5), the GDM risk increased by 58% with each 0.1-unit increase in AIP. Additionally, this tendency remained significant when the AIP was treated as a categorical variable (tertiles). As shown in Table 3, the risk of GDM gradually increased with elevated tertiles of the AIP across all models (*P* for trend < 0.001). Furthermore, there was a linear dose–response relationship between AIP and GDM risk in RCS (Fig. 2).

**Table 3:** Relationship between AIP and GDM risk in different models

	AIP*10		AIP tertile			
	OR, 95% CI	*P*-value	Q1 (OR, 95% CI)	Q2 (OR, 95% CI)	Q3 (OR, 95% CI)	*P*-trend
Model 1	1.77 (1.47–2.13)	< 0.001	1 (Ref)	2.38 (0.61–9.35)	9.85 (2.93–33.10)	< 0.001
Model 2	1.69 (1.39–2.05)	< 0.001	1 (Ref)	1.87 (0.45–7.67)	7.31 (2.10–25.46)	< 0.001
Model 3	1.67 (1.37–2.04)	< 0.001	1 (Ref)	1.80 (0.44–7.38)	6.67 (1.89–23.50)	< 0.001
Model 4	1.65 (1.33–2.04)	< 0.001	1 (Ref)	1.87 (0.43–8.05)	6.19 (1.63–23.53)	0.001
Model 5	1.58 (1.27–1.97)	< 0.001	1 (Ref)	2.39 (0.53–10.81)	6.52 (1.63–26.03)	0.002

Model 1: No adjusted

Model 2: Adjusted for Age + BMI + Nulliparity

Model 3: Adjusted for Age + BMI + Nulliparity + AST + ALT + GGT

Model 4: Adjusted for Age + BMI + Nulliparity + AST + ALT + GGT + FPG + Insulin + HOMA-IR

Model 5: Adjusted for Age + BMI + Nulliparity + AST + ALT + GGT + FPG + Insulin + HOMA-IR + TC + LDL + NAFLD

### Sensitivity analysis

We additionally carried out several sensitivity analyses to evaluate the robustness of our conclusions. Our first sensitivity analysis focused on participants with a pre-pregnancy BMI < 25 kg/m^2^, as shown in Table 4, a strong correlation was found between AIP and GDM risk even after accounting for other variables (OR 1.72, 95% CI 1.29–2.31). Another similar sensitivity analysis was conducted on participants without NAFLD, which also showed that AIP continued to have a positive correlation with the GDM risk even after accounting for other variables (OR 1.73, 95% CI 1.27–2.34) (Table 4). The sensitivity analysis indicated that our findings were reliable.

**Table 4:** Relationship between AIP and GDM risk in different sensitivity analyses

Variable	Model I (OR, 95% CI, *P*)	Model II (OR, 95% CI, *P*)
AIP*10	1.72 (1.29–2.31) < 0.001	1.73 (1.27–2.34) < 0.001
AIP tertile		
Q1	1 (Ref)	1 (Ref)
Q2	1.11 (0.15–8.21) 0.917	0.89 (0.11–6.88) 0.907
Q3	7.12 (1.44–35.14) 0.016	6.32 (1.19–33.65) 0.031
*P-*trend	0.007	0.009

Model I was a sensitivity analysis after excluding those with a pre-pregnancy BMI ≥ 25 kg/m^2^, *n* = 489. We adjusted for Age + Nulliparity + AST + ALT + GGT + FPG + Insulin + HOMA-IR + TC + LDL + NAFLD

Model II was a sensitivity analysis after excluding those with NAFLD, *n* = 475. We adjusted for Age + BMI + Nulliparity + AST + ALT + GGT + FPG + Insulin + HOMA-IR + TC + LDL

**Fig. 3:**
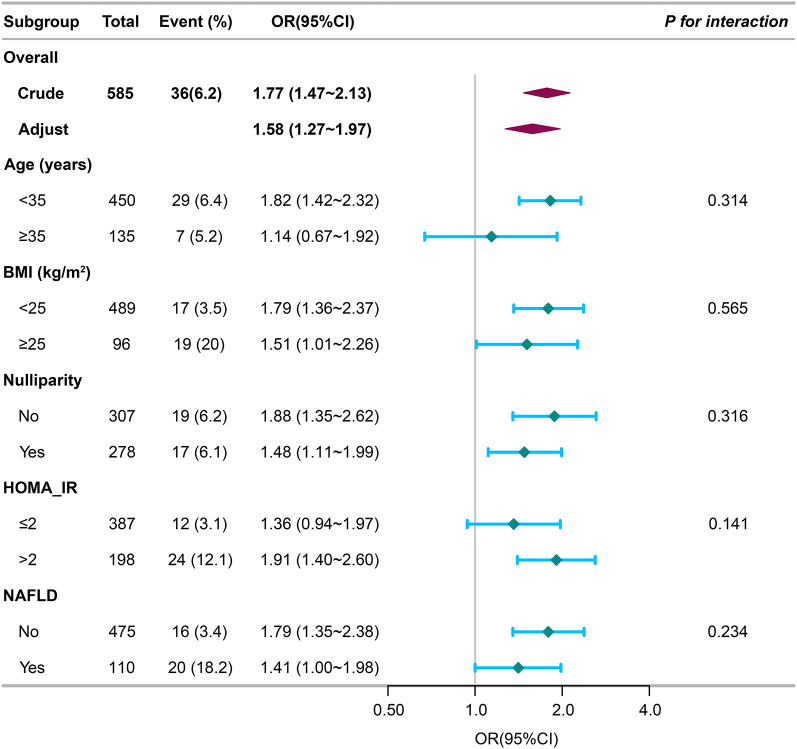
The forest plot for effect size of AIP on GDM in prespecified and exploratory subgroups. Each stratification was adjusted for Age + BMI + Nulliparity + AST + ALT + GGT + FPG + Insulin + HOMA-IR + TC + LDL + NAFLD, except the stratification factor itself

Furthermore, we conducted subgroup analyses to explore potential variables that could influence the connection between AIP and GDM risk. Age, BMI, nulliparity, HOMA-IR, and NAFLD were selected as stratification variables. As shown in Fig. 3, the mentioned potential confounding factors had no effect on the association between AIP and GDM risk. The subgroup analyses highlight the strengths of our results.

Moreover, we calculated the E-value for estimating the required degree of correlation between an unmeasured confounder and the risk of GDM using the results from Model 5. Surprisingly, the E-value's point estimate was 2.54 when the AIP correlated positively with the risk of GDM. It seems improbable that unmeasured confounding variables would have much effect on the robustness of our findings compared to previous research on GDM [19, 20]. Clearly, after conducting various sensitivity analyses, our findings are robust.

### ROC analysis

**Fig. 4:**
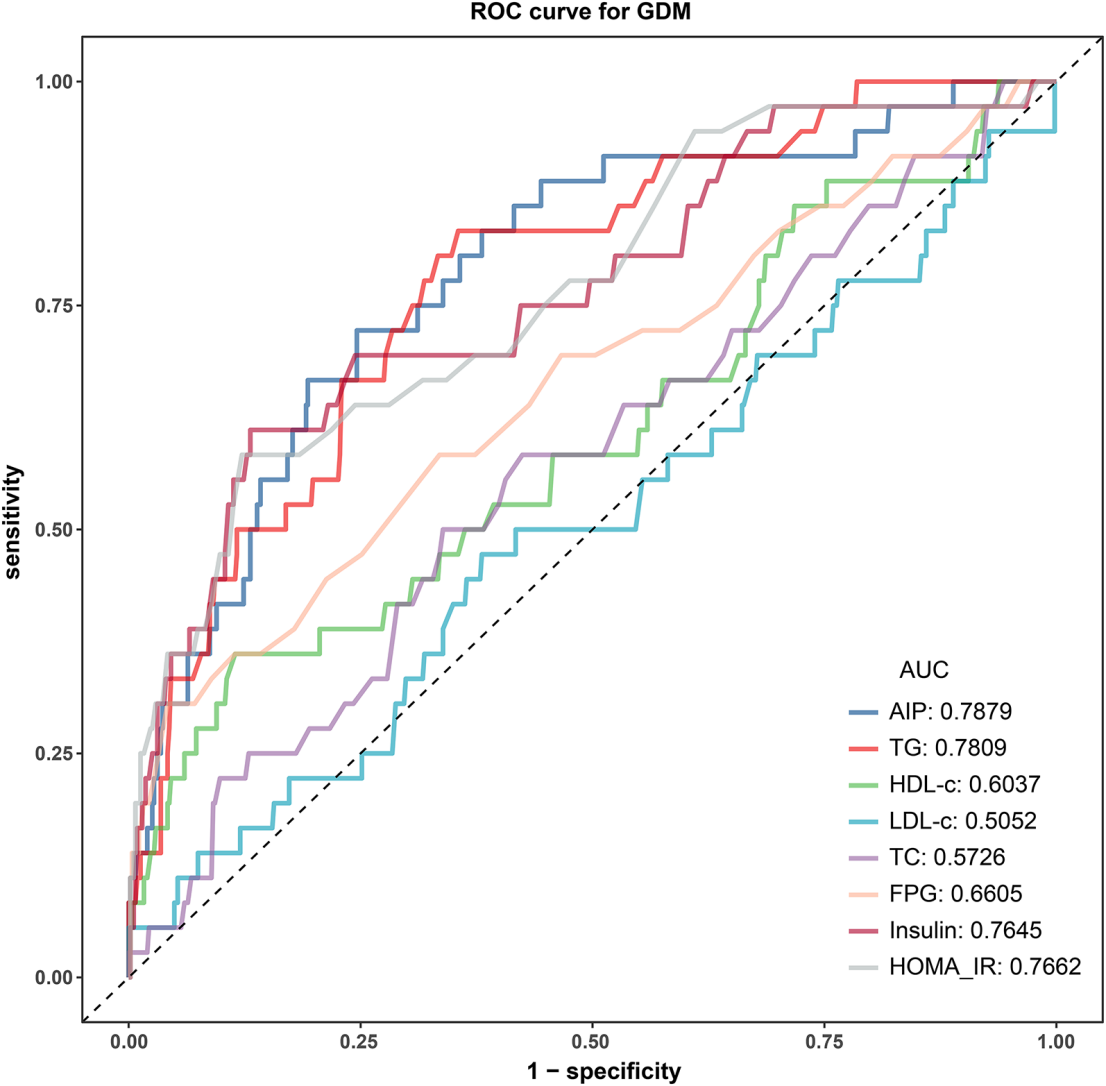
ROC curves for AIP to predict the risk of GDM in all participants.The results showed that the AUC of the AIP was 0.7879. Compared to TG, HDL, LDL, TC,FPG, insulin, and HOMA-IR, the AUC of the AIP for predicting GDM was the highest

The AIP's potential as a predictor for GDM was examined with ROC analysis, as presented in Table 5 and Fig. 4, and the AUC was 0.7879 (95% CI 0.7087–0.8671). Surprisingly, AIP demonstrated the highest AUC among various factors such as TG, HDL, LDL, TC, FPG, insulin, and HOMA-IR, suggesting its superior ability to predict GDM. Youden's index was utilized to identify the best threshold value of 0.3557 for AIP in predicting GDM, with a specificity of 75.41% and a sensitivity of 72.22%.

**Table 5:** Areas under the receiver operating characteristic curves (AUROC) for each evaluated parameters in identifying GDM

Variables	AUC (95% CI)	Best threshold	Specificity	Sensitivity	Youden index
AIP	0.7879 (0.7087–0.8671)	0.3557	0.7541	0.7222	0.4763
TG (mg/dL)	0.7809 (0.7049–0.8569)	121.5000	0.6448	0.8333	0.4781
HDL (mg/dL)	0.6037 (0.4976–0.7099)	49.2000	0.8852	0.3611	0.2463
LDL (mg/dL)	0.5052 (0.3997–0.6107)	77.5500	0.6193	0.4722	0.0915
TC (mg/dL)	0.5726 (0.4729–0.6723)	181.5000	0.6612	0.5000	0.1612
FPG (mg/dL)	0.6605 (0.5567–0.7643)	90.5000	0.9581	0.3056	0.2637
Insulin (μIU/mL)	0.7645 (0.6755–0.8535)	13.9000	0.8689	0.6111	0.4800
HOMA-IR	0.7662 (0.6803–0.8521)	2.7500	0.8780	0.5833	0.4613

## Discussion

In this study, 585 pregnant women were examined to investigate the connection between AIP and GDM risk in the Korean population. Our findings revealed that AIP was an independent risk factor for GDM in pregnant women and has a linear dose–response relationship with GDM. The sensitivity analysis findings additionally reinforce the positive relationship between AIP and GDM. Furthermore, our study showed that AIP has the ability to effectively predict GDM with an AUC of 0.7879 (0.7087–0.8671), and the optimal cut-off point of AIP for predicting GDM was 0.3557, with a sensitivity of 72.22% and a specificity of 75.41%. Notably, the AIP outperformed TG, HDL, LDL, TC, FPG, insulin, and HOMA-IR as predictors of GDM. Accordingly, AIP could serve as a viable non-invasive predictive tool for GDM.

Over the past few years, the prevalence of GDM among the overall Korean populace has increased, reaching 12.70% [21]. Our study found a lower GDM incidence of 6.15% compared to the documented rate. Since this study used stricter exclusion criteria (chronic liver disease,excessive alcohol consumption, and pre-gestational diabetes), as well as stricter diagnostic criteria for GDM (a two-step GDM test), all of which likely contributed to a lower incidence of GDM. Therefore, the observed incidence is considered acceptable. Nevertheless, it is worth highlighting that the incidence of GDM within this population remains at 6.15%. This underlines the significance of investigating possible extra risk factors for GDM.

Although the cause of GDM is not completely understood, maternal obesity, older maternal age, and specific ethnicities have been identified as being at high risk [22]. However, the relationship between dyslipidemia and GDM remains controversial [23, 24]. It is known that maternal dyslipidemia during pregnancy often surpasses the physiological range [25]. Lipid levels experience a slight rise in early pregnancy, followed by a considerable boost in later stages, and it is common to observe a lipid profile resembling the pathological features of metabolic syndrome [26–28], which can lead to atherosclerosis [29, 30]. These alterations are not indicative of a pathological state; rather, they are necessary physiological adjustments for the mother to fulfill the energy requirements the fetus. However, if these adaptive changes exceed a certain threshold, various pregnancy complications, such as GDM, can arise. Research has shown that pregnant individuals who have TG levels above 137 mg/dL encounter a 3.5-fold increase in GDM risk, with a 10% increase in GDM risk for every 20 mg/dL increase in TG levels [31]. However, Gao et al. [32] reported that remnant cholesterol (RC) was positively correlated with GDM, while there was no notable correlation between GDM and other lipoprotein cholesterols, such as TC, LDL, and HDL. This might be due to the use of composite lipid indexes, which exhibit greater sensitivity than a single lipid index. Boying Yin et al. [33, 34] also believed that, compared to single lipid indicators, composite lipid metrics exhibit greater sensitivity and a more precise reflection of dyslipidemia. AIP is as an unconventional lipid marker that integrates TG and HDL, indicating not just the TG to HDL ratio but also the lipoprotein particle size. It better reflects the pathogenicity of dyslipidemia compared to elevated TG or decreased HDL levels [35]. Several studies have found that AIP is not only related to CVD but can also predict the risk of T2DM [36]. There are limited studies that discuss the utilization of AIP in pregnant individuals, and to the best of our knowledge, no studies have examined how the AIP compares to other indicators in predicting the risk of GDM.

According to our study, AIP is identified as an independent risk factor for GDM in pregnant women, consistent with the findings of studies previously performed on the general population. A cross-sectional study of 10,099 American adults found that the prevalence of prediabetes and diabetes in women increased by 4.96 times for every one-unit increase in AIP [13]. In another study involving 100,069 adults from China, after adjusting for other confounding factors, AIP was found to have a positive and non-linear relationship with the incidence of prediabetes [37]. Another study on Chinese middle-aged and elderly people also found that AIP is an independent predictor of T2DM, and has a non-linear relationship with T2DM [38]. Different from them, our study found a linear relationship between AIP and GDM. This may be due to the fact that our study was specific to pregnant women, as lipid profiles during pregnancy show atherogenic alterations, which can lead to higher AIP levels in pregnant women. As reported by Bei Yin et al. [36], there is a nonlinear J-type relationship between AIP and T2DM. When AIP is greater than − 0.47, the increase in AIP is significantly correlated with the increased risk of T2DM, but when AIP is less than − 0.47, there is no significant correlation between AIP and T2DM. However, in our study, only one pregnant woman had AIP levels below − 0.47. Therefore, it is reasonable that our study found a linear relationship between AIP and GDM. So, our study population differed from those in previous studies. Furthermore, our study indicated that AIP is a more accurate predictor of GDM risk than TG, HDL, LDL, TC, FPG, insulin, and HOMA-IR are. Besides, the sensitivity analysis revealed that the correlation between AIP and GDM susceptibility remained noteworthy among Korean females with a BMI < 25 kg/m^2^ or without NAFLD. Moreover, there was a broader range of covariates in our study, such as nulliparity, AST, ALT, GGT, FPG, insulin, HOMA-IR, TC, LDL, and NAFLD, which are all linked with GDM risk. More importantly, we conducted additional sensitivity and subgroup analyses to bolster the reliability of the positive link between AIP and GDM risk. Clarifying the connection between AIP and GDM risk helps improve prevention strategies for GDM and offers significant predictors for the subsequent establishment of a GDM prediction model. Therefore, our findings that AIP is an independent risk factor for GDM and could serve as a focus for GDM prevention and treatment are not only a significant and clinically relevant finding but also further extends the AIP application population and have excellent clinical value.

Previous studies have indicated that TG, HDL, LDL, TC, FPG, insulin, and HOMA-IR are effective indicators for predicting the diabetes mellitus in the general population [39–41]. Our research utilized ROC analysis to to assess the ability of these parameters and AIP at 10–14 weeks of pregnancy to predict GDM. The findings indicated that the AIP exhibited strong predictive ability for GDM, with an AUC of 0.7879, surpassing that of traditional parameters. This indicates that AIP could serve as a more reliable predictor of GDM for pregnant women, particularly individuals with normal TG and HDL levels that may go unnoticed. Remarkably, we found that AIP was more accurate than HOMA-IR in predicting GDM, indicating its promise as a warning sign for IR early in the pregnancy and further demonstrating that AIP is a reliable predictor of GDM. Therefore, we recommend adding the calculation and analysis of AIP to the routine prenatal examination in early pregnancy. If a pregnant woman's AIP exceeds 0.3557 in the early pregnancy, she is at a high risk of developing GDM and should consider initiating GDM preventative strategies.

Although the exact biological mechanism underlying the relationship between GDM and AIP is unclear, the following ones can be suggested. The AIP is determined by merging the TG and HDL levels, both of which have a strong connection to the GDM risk. Early in pregnancy, increased levels of estrogen and IR may enhance the synthesis of lipids, mainly TG, in the liver [24]. Increased TG production causes increased levels of unbound fatty acids and toxic lipids, leading to insulin signaling changes in pancreatic α-cells and increased glucagon secretion [42]. High levels of glucagon are seen as a major contributor to hyperglycemia [43]. Furthermore, reduced HDL levels may impede cholesterol efflux, resulting in the accumulation of cholesterol in pancreatic *β*-cells and impaired insulin secretion, ultimately causing high blood glucose [44, 45]. Additionally, it can affect glucose homeostasis by reducing insulin sensitivity and the activity of AMP-activated protein kinase [46, 47]. Coincidentally, AIP is the logarithmic transformation of the ratio of TG to HDL, which has higher sensitivity to diseases compared to individual TG and HDL. Moreover, it reflects the equilibrium of real TG and HDL levels, helping to evaluate the directionality of cholesterol transportation in the vascular pool and better capturing the impact of lipid effects [10]. Interestingly, AIP is also notably linked to the rate of HDL esterification and the size of lipoprotein particles, both of which are predictors of IR [48, 49]. Surprisingly, AIP is also a direct independent predictor of IR [36]. These hypothesized mechanisms provide a pathophysiological basis for the correlation observed between AIP and GDM.

Our study has the following advantages. Most notably, we have effectively confirmed an independent and positive correlation between early pregnancy plasma AIP levels and GDM for the first time. Second, our research offers an AUROC value and the best threshold for predicting GDM early using the AIP. Third, we minimized residual confounding factors through strict statistical adjustment. Fourth, we carried out a variety of sensitivity analyses for assessing the reliability of our conclusions, such as transforming the AIP into a categorical factor, reevaluating the link between AIP and GDM by removing individuals with a BMI ≥ 25 kg/m^2^ and NAFLD, conducting subgroup examinations, and determining E-values to investigate potential unmeasured variables.

However, it is important to recognize some limitations of our research. First, our results should be confirmed in various ethnic populations due to potential differences in the connection between AIP and GDM based on race. Second, our study was a secondary analysis, and even after adjusting for possible confounders, there could inevitably be unmeasured or uncontrolled confounders. As a workaround, we have calculated the E-value based on our fully model to quantify the magnitude of association required by an unmeasured confounding factor. We find that the presence of unmeasured confounding variables is unlikely to impact the reliability of the results. Another limitation is that AIP was collected only once at 10–14 weeks of gestation, without considering its evolution with gestational age [50]. Therefore, future designs of our research may include capturing additional confounding variables and collect alterations in TG and HDL during follow-up. We will also intend to investigate how well our results hold in various populations.

## Conclusion

In summary, this research revealed that the AIP at 10–14 weeks of gestation has an independent and positive association with GDM, and could be used as a predictor for GDM in the Korean population. According to the threshold analysis, it is important for pregnant women to maintain an AIP below 0.36. Therefore, AIP could serve as an early screening and monitoring tool to identify pregnant women at high risk of GDM and optimize GDM prevention strategies.

### Supplementary Information

Supplementary Material 1.

## Data Availability

The dataset supporting the conclusions of this article is available in the [PLos one] repository, [unique persistent identifier and hyperlink to dataset in 10.1371/journal.pone.0221400].

## Abbreviations

AIP: Atherogenic index of plasma
GDM: Gestational diabetes mellitus
BMI: Body mass index
AST: Aspartate aminotransferase
ALT: Alanine aminotransferase
GGT: Gamma-glutamyl transferase
TC: Total cholesterol
TG: Triglyceride
HDL: High-density lipoprotein cholesterol
LDL: Low-density lipoprotein cholesterol
NAFLD: Nonalcoholic fatty liver disease
FPG: Fasting plasma glucose
IR: Insulin resistance
HOMA-IR: Homeostasis model assessment-insulin resistance
RCS: Restricted cubic splines
ROC: Receiver operating characteristic
AUC: Area under the curve
OR: Odds ratio
CI: Confidence interval
Ref: Reference
