# Supplementary material for: Association between atherogenic index of plasma and gestational diabetes mellitus: a prospective cohort study based on the Korean population
Source: Cardiovasc Diabetol. 2024 Jul 5;23:237. doi: 10.1186/s12933-024-02341-9 (PMC11227226; doi:10.1186/s12933-024-02341-9)
Supplement: Supplementary file 1 — Supplementary Material 1. [file 12933_2024_2341_MOESM1_ESM.docx]

**Table S1** Comparison of basic characteristics between included and excluded populations.

| **Characteristics** | **Total**  **(*n*=623)** | **Included population**  **(*n*=585)** | **Excluded population**  **(*n*=38)** | ***P*-value** |
| --- | --- | --- | --- | --- |
| Age (years) | 32.07 ± 3.87 | 32.06 ± 3.76 | 32.24 ± 5.27 | 0.783 |
| BMI (kg/m^2^) | 22.08 ± 3.50 | 22.02 ± 3.49 | 23.03 ± 3.52 | 0.089 |
| Nulliparity |  |  |  | 0.355 |
| No | 324 (52.01) | 307 (52.48) | 17 (44.74) |  |
| Yes | 299 (47.99) | 278 (47.52) | 21 (55.26) |  |
| AST (IU/L) | 16.00 (14.00-20.00) | 16.00 (14.00-20.00) | 17.50 (14.75-22.75) | 0.329 |
| ALT (IU/L) | 11.00 (8.00-15.00) | 11.00 (8.00-15.00) | 13.50 (8.75-17.25) | 0.337 |
| GGT (IU/L) | 12.00 (10.00-15.00) | 12.00 (10.00-15.00) | 12.50 (11.00-18.50) | 0.407 |
| TC (mg/dL) | 173.12 ± 27.29 | 172.84 ± 27.10 | 182.28 ± 32.50 | 0.149 |
| TG (mg/dL) | 119.46 ± 48.23 | 118.80 ± 47.54 | 141.17 ± 64.89 | 0.053 |
| HDL (mg/dL) | 64.96 ± 13.59 | 64.97 ± 13.54 | 64.71 ± 15.32 | 0.936 |
| LDL (mg/dL) | 84.15 ± 21.88 | 83.99 ± 21.70 | 89.34 ± 27.28 | 0.307 |
| NAFLD |  |  |  | 0.732 |
| No | 505 (81.06) | 475 (81.20) | 30 (78.95) |  |
| Yes | 118 (18.94) | 110 (18.80) | 8 (21.05) |  |
| FPG (mg/dL) | 77.11 ± 9.67 | 76.97 ± 9.72 | 82.00 ± 6.07 | 0.034 |
| Insulin (μIU/mL) | 8.40 (5.40-11.67) | 8.40 (5.40-11.50) | 9.70 (7.10-13.30) | 0.100 |
| HOMA-IR | 1.50 (1.00-2.30) | 1.50 (1.00-2.30) | 2.00 (1.50-2.60) | 0.065 |
| GDM |  |  |  | 0.210 |
| No | 571 (93.61) | 549 (93.85) | 22 (88.00) |  |
| Yes | 39 ( 6.39) | 36 (6.15) | 3 (12.00) |  |
| AIP | 0.22 (0.11-0.37) | 0.22 (0.11-0.37) | 0.32 (0.20-0.44) | 0.106 |

Values were expressed as mean (standard deviation) or median (interquartile range) or *n* (%)

Abbreviations: BMI pre-pregnancy body mass index; AST, aspartate aminotransferase; ALT, alanine aminotransferase; GGT, gamma-glutamyl transferase; TC, total cholesterol; TG, triglyceride; HDL, high-density lipoprotein cholesterol; LDL, low-density lipid cholesterol; NAFLD, nonalcoholic fatty liver disease; FPG, fasting plasma glucose; HOMA-IR, homeostasis model assessment-insulin resistance; GDM, gestational diabetes mellitus; AIP, atherogenic index of plasma.
